# Microbiological quality of kitchens sponges used in university student dormitories

**DOI:** 10.1186/s12889-020-09452-4

**Published:** 2020-08-31

**Authors:** Tareq M. Osaili, Reyad S. Obaid, Klaithem Alowais, Rawan Almahmood, Moza Almansoori, Noora Alayadhi, Najla Alowais, Klaithem Waheed, Dinesh Kumar Dhanasekaran, Anas A. Al-Nabulsi, Mutamed Ayyash, Stephen J. Forsythe

**Affiliations:** 1 Department of Clinical Nutrition and Dietetics, College of Health Sciences, University of Sharjah, P. O. Box 27272, Sharjah, United Arab Emirates; 2 Research Institute for Medical and Health Sciences, University of Sharjah, P. O. Box 27272, Sharjah, United Arab Emirates; 3 Department of Nutrition and Food Technology, Faculty of Agriculture, Jordan University of Science and Technology, P.O. Box 3030, Irbid, 22110 Jordan; 4 Department of Food, Nutrition and Health, College of Food and Agriculture, United Arab Emirates University (UAEU), Al Ain, Abu Dhabi, United Arab Emirates; 5 Foodmicrobe.com, Adams Hill, Keyworth, Nottingham, NG12 5GY UK

**Keywords:** Sponges, Kitchen, Cleaning, Dormitories, Storage, *Enterobacteriaceae*, Antibiotic resistance

## Abstract

**Background:**

Kitchen sponges are a major source of cross-contamination as they can transfer foodborne pathogens, infectious agents and spoilage causing microorganisms to food contact surfaces. Several studies have revealed that university students adopt poor practices regarding food safety, hygiene, and the handling of kitchen cleaning equipment.

**Methods:**

A total of fifty kitchen sponges were collected along with a questionnaire addressing social demographics and kitchen sponge usage by students living at the University of Sharjah dormitories. The effect of storage (3 and 10 days) on the microbial population of kitchen sponges at room temperature (21 °C) was assessed. *Enterobacteriaceae* isolated from sponges were identified and their antibiotic resistance determined.

**Results:**

Student responses revealed that kitchen sponges used to clean food contact surfaces were also used to clean the oven (32%), sink (26%), refrigerator (10%), and to clean spills on the floor (4%). Kitchen sponges contained high counts of mesophilic aerobic bacteria (7.9 log_10_/cm^3^*)*, coliform (7.2 log_10_/cm^3^), *Enterobacteriaceae* (7.3 log_10_/cm^3^) and yeasts and molds (7.0 log_10_/cm^3^). After storage of the sponges at room temperature (21 °C) for 3 and 10 days, the number of mesophilic aerobic bacteria, coliform, *Enterobacteriaceae* and yeasts and molds decreased by 0.4 and 1.3 log_10_/cm^3^, 0.7 and 1.4 log_10_/cm^3^, 0.4 and 1.1 log_10_/cm^3^, and 0.6 and 1.3 log_10_/cm^3^, respectively. The most frequently isolated *Enterobacteriaceae* were *Enterobacter cloacae* (56%) and *Klebsiella oxytoca* (16%)*.* All *E. cloacae* isolates were resistant to amoxicillin, cefalotin, cefoxitin and cefuroxime axetil.

**Conclusions:**

This study showed that students living in dormitories lacked good hygienic practices and were at increased risk of food poisoning. Kitchen sponges were highly contaminated with potentially pathogenic bacteria which could be transferred from the general kitchen environment to food contact surfaces and consequently lead to food contamination.

## Background

Kitchen sponges are one of the main cleaning tools used to clean kitchen utensils and surfaces such as kitchen utensils cutting boards, sinks, oven tops and refrigerators [1, 2]. However, during cleaning food residues may adhere to the sponge surface and damp sites such as sink areas can act as further microbial reservoirs that can contaminate the sponges during their use [3]. Subsequent poor handling, storage or improper disinfection of kitchen sponges will lead to further microbial growth at room temperature [4]. Consequently, kitchen sponges are a major source of cross-contamination as they can transmit foodborne pathogens, infectious agents and spoilage causing microorganisms to food contact surfaces [5, 6].

A study conducted in 10 kitchens in the United States of America found that 33 and 67% of the tested sponges were positive for *Escherichia coli* and fecal coliforms, respectively [7]. Furthermore, Enriquez et al. [8] found that 15.4% of sponge samples taken from households were contaminated with *Salmonella* spp. Further studies have shown that kitchen sponges collected from domestic kitchens were contaminated with *Campylobacter* spp., *Enterobacter cloacae*, *E. coli*, *Klebsiella* spp., *Proteus* spp., *Salmonella* spp., *Acinetobacter*, *Moraxella,* and *Staphylococcus* spp. [9–14]. In similar studies, kitchen sponges were found to harbor high counts of aerobic mesophilic bacteria, coliforms, *Enterobacteriaceae*, yeasts and molds due to poor kitchen sponge sanitization practices [4, 8, 15–18].

Coliforms are a general group of bacteria within the *Enterobacteriaceae* which have been used as indicator microorganisms for hygiene monitoring purposes. Coliforms can be found in the kitchen due to inadequate disinfection procedures, inappropriate sanitary and hygienic practices, and contamination by raw products, and cross-contamination from contaminated food [19]. In a hygiene study of a household, kitchen sponges had the second highest load coliforms, the highest being drain traps [20].

Beside the significant health, social, and economic impact of microbial infections [21], the increase in antibiotic resistant bacteria is a serious threat to human health which may result in incurable infections and fatality in both developed and developing regions [22]. *Enterobacteriaceae* are potential causes of serious infections and are increasingly resistant to antibiotics. A major concern with *Enterobacteriaceae* is the production of ESBLs leading to multidrug resistance and creating pan-resistant bacterial strains [23]. ESBL encoding *Enterobacteriaceae* are usually found in the hospital setting but are becoming prevalent in the community as well [24]. Widespread recovery of multidrug-resistant *Enterobacteriaceae* from chicken meat would lead to restricted treatment options if infections occur [21].

Several studies of university students have shown that they adopt poor practices regarding food safety, hygiene, and the handling of kitchen cleaning tools such as sponges [25–27]. Consequently, they increase their risk to foodborne illnesses [25–27]. The aims of this study were to (i) explore the microbial quality and usage of kitchens sponges used at the University of Sharjah student dormitories, (ii) assess the effect of storage on the microbial populations in the sponges, and (iii) identify any *Enterobacteriaceae* isolated from sponges and determine their antibiotic resistance profiles.

## Methods

### Sample collection and kitchen sponge usage questionnaire

The researchers visited the female dormitories (two locations) at the University of Sharjah twice a week on different days between 8:00 am-12:00 pm randomly without prior notification and explained the objectives of study to the students who were available in the rooms during the visits. Usually, 1–2 students stay in each room which consists of a bedroom, small kitchen and bathroom. Students who agreed to participate in the study signed a consent form. Fifty students from the two locations agreed to participate in the study.

Kitchen sponges used in the kitchens were collected from students who agreed to participate in the study (*n* = 50) using sterilized forceps. Only one sponge was used in each room; students share the same sponge in the two-student rooms. The temperature of the rooms in the dormitories were maintained at 21 °C and there were no windows near to the kitchens. The sponges were kept on the sinks in the kitchens and were not exposed to direct sunlight. The collected sponges were put separately into sterilized plastic bags and transferred within 30 min to Nutrition and Food laboratory (Research Institute for Medical and Health Sciences, University of Sharjah) in an ice box for analysis. In addition, a questionnaire designed by the investigators for this study was completed by the participants (*n* = 50). The questionnaire was consisted of two parts; the first part included questions on their socio-demographic profile, whereas the second part included questions on their kitchen sponge usage (available in the supplementary files).

### Sample preparation for analysis

The materials of the kitchen sponges collected from students’ dormitories were polyester (soft yellow side) and polyurethane (abrasive side). Each sponge was coded and cut into three parts using a sterile knife. The average dimensions of the sponge parts were 6.6 cm × 3.2 cm × 3.3 cm. The first part of the sponge was analyzed microbiologically within 1 h from receipt. The second and third parts of the sponge were kept separately at ambient temperature (21 °C ± 1) and a relative humidity of 59% ± 2 for 3 and 10 days in a sterile un-covered plastic container.

### Weight and water activity measurements

The weight and water activity (a_w_) (Labmaster aw, Novasina AG, Switzerland) of the inner and external parts of the sponge samples (W 2 cm x H 1.5 cm) at time 0, 3 and 10 days before microbiological analysis of the samples were measured. The average value of the readings was used.

### Microbiological analysis

The sponge samples were added to 100 ml of 0.1% peptone water (HiMedia Laboratories, India) in a sterilized stomacher bag, and put in the mixing machine (blender EASYMIX, bioMérieux, France) for 2 min. After that, decimal dilutions were made and 1 or 0.1 ml from appropriate dilution was plated on duplicate on violet red bile glucose agar (VRBGA, HiMedia) for enumeration of *Enterobacteriaceae*, violet red bile agar (VRBA, HiMedia) for enumeration of coliforms, total plate count agar (TPCA, HiMedia) for enumeration of mesophilic aerobic bacteria, and Sabouraud dextrose agar (SDA, Mast Group Ltd., UK) for enumeration of yeasts and molds, by using the pour plate technique. VRBGA and TPCA were incubated aerobically for 48 h at 32 °C, while VRBA was kept aerobically at 37 °C for 48 h. The SDA plates were kept aerobically at 21 °C for 5 days. Unused sponges (*n* = 3) representing the same brands of the sponge samples used in the study were purchased from local markets and used as negative controls.

### Identification of *Enterobacteriaceae* isolates

One typical *Enterobacteriaceae* colony representing each sponge sample (pink or purple color colonies) was picked from VRBGA and inoculated into 10 ml tryptone soya broth (HiMedia). The tubes were mixed and incubated in 32 °C for 24 h before being streaked on nutrient agar HiMedia) and incubated for 24 h at 32 °C. Isolates were identified using the VITEK 2 GN ID card (bioMerieux, Marcy-l'Étoile, France) as according to the manufacturer’s instructions.

### Antibiotic resistance test

The antibiotic susceptibility of *Enterobacteriaceae* isolates was determined using the VITEK 2 (bioMerieux) using the Gram-negative susceptibility card (AST-N215). The card tested the susceptibility of the isolates to ampicillin, amoxicillin, piperacillin/tazobactam, cefalotin, cefuroxime, cefuroxime axetil, cefoxitin, cefpodoxime, cefotaxime, ceftazidime, imipenem, meropenem, gentamicin, tobramycin, ciprofloxacin, norfloxacin, tetracycline, nitrofurantoin, and trimethoprim/sulfamethoxazole.

### Statistical analysis

The data collected from the questionnaire, as well as the identification and antibiotic resistance profile of the *Enterobacteriaceae* isolates were analyzed by using IBM SPSS Statistics 25.0 software for descriptive statistical mean, percentage, standard deviation, and frequency. The differences in the microbial populations in the sponge samples during storage were tested with one-way analysis of variance (ANOVA) and post-hoc analysis by Tukey. Statistical significance at *P*-value < 0.05 was considered to be a significant level.

## Results

The results of the questionnaire study (Table 1) showed that more than half (66%) of the participants were medical students (medicine, dentistry, pharmacy and health sciences colleges). The respondents were mostly from the first and fourth year, while fifth- and second- year students were the least. Two thirds of the respondents stated that the sponges were used by two students, less than one third (30%) were used by one student, and a small percentage (4%) by three students. More than half the participants (58%) had used the sponge for less than one month, 32% used the sponge for two months, and 10% had used the sponge for more than 2 months. Seventy percent of participants do not sanitize their sponges. The main use of the sponges was for cleaning utensils (94%) and cooking pots (90%). About two thirds of the collected sponges were used to clean cutting boards. One third of the collected sponge samples (32%) were used to clean the oven, while 26 and 30% of the collected sponges are used to clean sink and surfaces around the sink, respectively. A very low percentage of 4 and 10% of the students clean spills on kitchen floor and interior/exterior surfaces of the refrigerator using the same sponge that is used to clean food utensils.

**Table 1:** Characteristics and responses of students living in university dormitories to questions on kitchen sponge usage

Questions	Frequency	Percentage
**College**		
Medical	33	66%
Nonmedical	17	34%
**Year**		
First	18	36%
Second	4	8%
Third	10	20%
Fourth	13	26%
Fifth	5	10%
**How many students are using the sponge?**		
One	15	30%
Two	33	66%
Three	2	4%
**How long have you been using this sponge?**		
Less than one month	29	58%
Two months	16	32%
Three months	2	4%
More than three months	3	6%
**Do you clean or sanitize the sponge?**		
Yes	15	30%
No	35	70%
**Do you use this sponge in cleaning plates and silver utensils?**		
Yes	47	94%
No	3	6%
**Do you use this sponge in cleaning cooking pot?**		
Yes	45	90%
No	5	10%
**Do you use this sponge in cleaning cutting boards?**		
Yes	32	64%
No	18	36%
**Do you use this sponge in cleaning stove?**		
Yes	16	32%
No	34	68%
**Do you use this sponge in cleaning sink?**		
Yes	13	26%
No	37	74%
**Do you use this sponge in cleaning surfaces beside the sink?**		
Yes	15	30%
No	35	70%
**Do you use this sponge in cleaning spills on the kitchen floor?**		
Yes	2	4%
No	48	96%
**Do you use this sponge in cleaning interior and exterior surfaces of the refrigerator?**		
Yes	5	10%
No	45	90%

The mean weight of sponges (*n* = 6) declined during storage; 6.22 g (±1.74) in 0 day, 2.72 g (±0.54) in 3 days, and 2.59 g (±0.57) in 10 days. The mean a_w_ value of sponges was 0.93 (±0.18) in 0 day. This decreased to 0.57 (±0.16) and 0.55 (±0.06) after 3 and 10 days of storage.

**Fig. 1:**
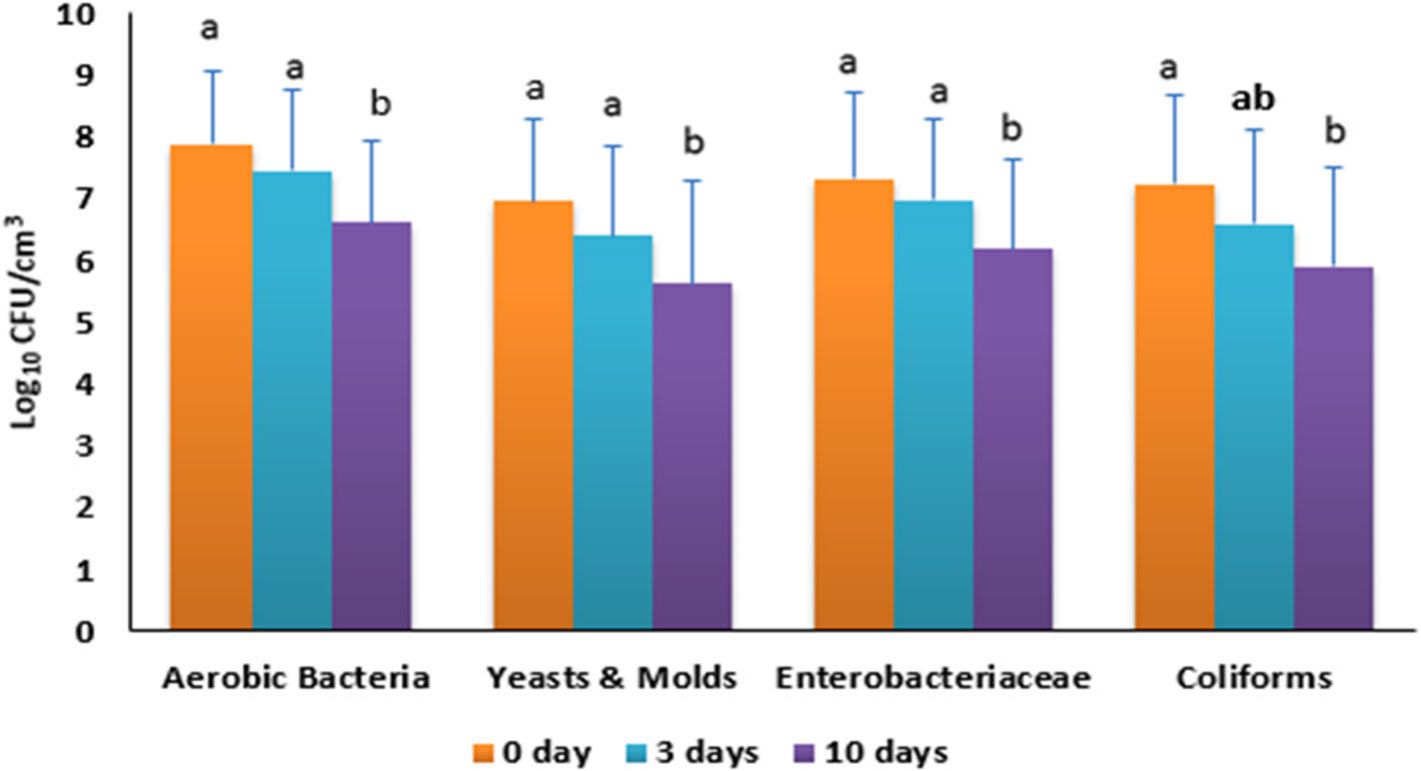
Microbial populations (log10 CFU/cm^3^) in kitchen sponge samples collected from student dormitories during storage at room temperature (21 °C) for 0, 3 and 10 days. Different letters per tested group indicate significant differences among the microbial populations (*P* < 0.05)

The viable counts of mesophilic aerobic bacteria, yeasts and molds, *Enterobacteriaceae,* and coliforms in sponge samples during storage at room temperature (21 °C) for 0, 3 and 10 days are shown in Fig. 1. The populations of the tested microorganisms in the sponge samples decreased as the storage time increased. There was no significant difference between the populations of the microorganisms from sponge samples at time 0 and after 3 days of storage, while significant differences were found on day 10 of storage. Mesophilic aerobic bacteria counts decreased after 3 and 10 days of storage by 0.4 and 1.3 log_10_/cm^3^ respectively. Yeasts and molds populations decreased after 3 and 10 days of storage by 0.6 and 1.3 log_10_/cm^3^ respectively. *Enterobacteriaceae* populations decreased after 3 and 10 days of storage by 0.4 and 1.1 log_10_/cm^3^ respectively. Similarly, coliform populations decreased after 3 and 10 days of storage by 0.7 and 1.4 log_10_/cm^3^ respectively. For the negative control sample, mesophilic aerobic bacteria and yeasts and molds populations were 2.46 and 1.80 log_10_/cm^3^ respectively. No *Enterobacteriaceae* or coliforms were detected in the samples.

*E. cloacae* were the most prevalent (56%) *Enterobacteriaceae* isolated (Table 2). *K. oxytoca* was the next most frequently isolated organism (16%), with *E. aerogenes* and *K. pneumoniae* being recovered from 6% of the samples. *Raoultella ornithinolytica* and *Serratia marcescens* were less frequently isolated (4%). Other *Enterobacteriaceae* were *Lelliottia amnigena*, *Pantoea* spp., *Kluyvera intermedia* and *Cedecea davisae* (2%).

**Table 2:** Identification of *Enterobacteriaceae* isolates from kitchen sponge samples

Microorganisms	Frequency	Percentage	Probability
*Cedeceadavisae*	1	2.0%	92%
*Enterobacter aerogenes*	3	6.0%	89–99%
*Enterobacter cloacae*	28	56.0%	89–99%
*Klebsiella oxytoca*	8	16.0%	95–99%
*Klebsiella pneumoniae*	3	6.0%	92–99%
*Kluyvera intermedia*	1	2.0%	88%
*Lelliottia amnigena*	1	2.0%	91%
*Pantoea spp*	1	2.0%	98%
*Raoultella ornithinolytica*	2	4.0%	94–95%
*Serratia marcescens*	2	4.0%	91–99%
Total	50	100%	

Table 3 shows the antibiotic resistance of the *Enterobacteriaceae* isolates. The antibiograms varied from no resistance, to strains that showed resistance to 9 antibiotics. All *E. cloacae* isolates (*n* = 28) were resistant to amoxicillin, cefalotin, cefuroxime, cefoxitin and cefuroxime axetil. Only one isolate was resistant to cefpodoxime. *E. aerogenes* was resistant to ampicillin, amoxicillin, cefalotin, cefuroxime, cefoxitin, cefuroxime axetil and cefpodoxime. The *C. davisae* isolate was resistant to ampicillin, amoxicillin, cefalotin and cefoxitin. *Klu. intermedia* was susceptible to all antibiotics. All *K. pneumoniae* isolates (*n* = 3) showed high resistance to ampicillin and one isolate showed a resistance to amoxicillin. *K. oxytoca* is resistant to ampicillin, amoxicillin, cefalotin, cefuroxime, cefuroxime axetil, cefoxitin, cefpodoxime, cefotaxime, ceftazidime. *L. amnigena* and *Pantoea* spp. showed resistance to amoxicillin, cefalotin and cefoxitin, while resistance to cefuroxime and cefuroxime axetil was observed in *L. amnigena*. *R. ornithinolytica* and *S. marcescens* were resistant to amoxicillin, cefalotin, and cefoxitin also, *R. ornithinolytica* is resistant to ampicillin and *S. marcescens* resistant to cefuroxime axetil.

**Table 3:** Antibiotic resistance of *Enterobacteriaceae* isolates from kitchen sponge samples collected from university student dormitories*

	Frequency	Ampicillin	Amoxicillin	Piperacillin/ Tazobactam	Cefalotin	Cefuroxime	Cefuroxime Axetil	Cefoxitin	Cefpodoxime	Cefotaxime	Ceftazidime	Nitro furantoin
*Cedece adavisae*	1	1(100%)**	1(100%)	0%	1(100%)	0%	0%	1(100%)	0%	0%	0%	0%
*Enterobacter aerogenes*	3	3(100%)	2(66.6%)	0%	3(100%)	3(100%)	3(100%)	3(100%)	1(33.3%)	0%	0%	0%
*Enterobacter cloacae*	28	0%	28(100%)	0%	28(100%)	28(100%)	28(100%)	28(100%)	1(3.5%)	0%	0%	0%
*Klebsiella oxytoca*	8	6(75%)	3(37.5%)	0%	4(50%)	2(25%)	2(25%)	4(50%)	2(25%)	1(12.5%)	1(12.5%)	0%
*Klebsiella pneumoniae*	3	3(100%)	1(33.3%)	0%	0%	0%	0%	0%	0%	0%	0%	0%
*Kluyvera intermedia*	1	0%	0%	0%	0%	0%	0%	0%	0%	0%	0%	0%
*Lelliottia amnigena*	1	0%	100%	0%	100%	100%	100%	100%	0%	0%	0%	0%
*Pantoea spp*	1	0%	100%	0%	100%	0%	0%	100%	0%	0%	0%	0%
*Raoultella ornithinolytica*	2	1(50%)	100%	0%	100%	1(50%)	0%	100%	0%	0%	0%	0%
*Serratia marcescens*	2	0%	100%	0%	100%	1(50%)	1(50%)	100%	0%	0%	0%	1(50%)

* All of the isolates were sensitive to imipenem, meropenem, gentamicin, tobramycin, ciprofloxacin, norfloxacin, tetracycline, and trimethoprim/sulfamethoxazole

** Number of isolates (%)

## Discussion

Good hygienic practices in the kitchen should include the separation of raw from ready to eat food and ensure work surfaces and utensils are clean to reduce the transfer of foodborne pathogens as a result of cross-contamination. Disposable wipes are a suitable alternative, but there may be financial reasons for using reusable sponges or cloths. The use of sponge and their microbial contamination reflects the food safety practices of students. The results of the current study showed that students have insufficient knowledge on the proper use of reusable kitchen sponges as they reported using them for multiple purposes. Cleaning the refrigerator and sink with the same sponges that are used to clean food contact surfaces is the probable reason for the high microbial load of the sponges. A previous study reported that the sink area harbored one of the highest levels of microbial contamination, including coliform, fecal coliform and *Enterobacteriaceae* [25].

The student practices increase the risk of cross-contamination with pathogenic organisms (environmental and foodborne) being transferred to food contact surfaces such as utensils and then on to ready to eat food, leading to infection. Previous studies conducted among university students have also shown that students had poor food safety knowledge on cross-contamination [27, 28].

The time intervals chosen to store sponges (3 and 10 days) was to mimic the length of student absence for weekends (3 days) or study break (10 days). The decrease in mesophilic aerobic bacteria, yeasts and molds, *Enterobacteriaceae* and coliform counts was due to the storage of sponges at room temperature (21 °C) leading to the lack of moisture for any further microbial survival/growth.

A number of the *Enterobacteriaceae* isolated are recognized bacterial pathogens (Table 2). *E. aerogenes* is associated with nosocomial infections of immunocompromised patients [29, 30]. *E. cloacae* can cause opportunistic infection, such as meningitis [31]. It is one of the predominant bacteria isolated from domestic kitchens and student sponge samples [3, 5, 32]. *K. oxytoca*, second highest frequency isolate in this study, has previously been isolated from domestic kitchen sponges [5] and leafy green vegetables [33]. This bacterium can cause septicemia, pneumonia and urinary tract infection [34]. *K. pneumoniae*, can cause serious community-acquired pneumonia [35], and has been isolated from meat and meat contact surfaces [36], leafy green vegetables [33] as well as kitchen sponges [5]. The pathogenicity of *R. ornithinolytica* is uncertain but it has been associated with fish and causes scombroid syndrome [37]. The symptoms of which includes vomiting and flushing [38]. *S. marcescens* has been isolated from cooked refrigerated pork meat and can cause pneumonia and urinary tract infection [39, 40]. Infection can be acquired through ingestion of contaminated food [41]. *L. amnigena* is considered an indicator of food contamination [42, 43]. *E. cloacae, K. pneumoniaa*, and *Pantoea* spp. have been isolated from leafy green vegetables [33, 44] which could explain their recovery from sponge used with cutting board. *Pantoea* spp. is mainly an environmental organism and can cause urinary tract infection and diarrhea [45]. *C. davisae* and *Klu. intermedia* are mainly present in respiratory tract [33, 46]. Kluyvera sp. are found in environmental sources such as food, water, flies and sink [47].

The emergence of multidrug-resistant *Enterobacteriaceae* is a major concern to human health. In the present study, the antibiograms of the *Enterobacteriaceae* isolates showed that all *E. cloacae* isolates (*n* = 28) were resistant to amoxicillin, cefalotin, cefuroxime, cefoxitin and cefuroxime axetil while only one isolate was resistant to cefpodoxime. Similar to our findings, *E. cloacae* isolates from healthy broilers were reported to be resistant to amoxicillin [48] and those from green leafy vegetables were resistant to cephalothin, cefuroroxime, cefoxitin, amipicillin, and amoxicillin-clavulanate [49]. The increasing antibiotic resistance of pathogenic bacteria is a major public health issue as that will increase foodborne illnesses and narrow treatment choices [50].

## Conclusion

In conclusion, female students live in dormitories at the University of Sharjah showed a lack of general knowledge regarding good hygienic practices. Kitchen sponges which were primarily for cleaning food contact surfaces, were often used for other general purposes and kept for prolonged periods of time. Leaving the sponges at room temperature for a few days did not reduce the microbial load of the sponges. These sponges were colonized by potentially pathogenic bacteria, which encoded for various antibiotic resistances. Students need to be reminded of good hygienic practices in order to reduce the risk of contaminating ready to eat food from raw foods, and the kitchen environment including the floor. This study has some potential limitations; the sample size was small and the study was conducted with female students as the data were collected by females who were not allowed to enter males dormitories; thus, further research is needed to include more students from both genders from other universities.

## Supplementary information

**Additional file 1.** Questionnaire on the use of the kitchen sponge at the dormitories. The file contains the questions used in the questionnaire.

## Data Availability

The datasets used during the present study are available from the first author on reasonable request.

## Abbreviations

ESBLs: Extended-Spectrum β-lactamases
