# Supplementary material for: Microbiological quality of kitchens sponges used in university student dormitories
Source: BMC Public Health. 2020 Aug 31;20:1322. doi: 10.1186/s12889-020-09452-4 (PMC7460773; doi:10.1186/s12889-020-09452-4)
Supplement: Supplementary file 1 — Additional file 1. Questionnaire on the use of the kitchen sponge at the dormitories. The file contains the questions used in the questionnaire. [file 12889_2020_9452_MOESM1_ESM.docx]

**Questionnaire on the use of the kitchen sponge at the dormitories**

**Sample number:**

**College:**

**Year:**

1. How many students are using the sponge?
2. One
3. Two
4. Three
5. How long have you been using the sponge?
6. Less than one month
7. Two months
8. Three months
9. More than three months
10. Do you clean or sanitize the sponge?
11. No
12. Yes

If yes specify how: _____________

1. Do you use this sponge in cleaning?

a. Plates and silver utensils: yes, no

b. Cooking pot: yes, no

c. Stove: yes, no

d. Cutting boards: yes, no

e. Sink: yes, no

f: Surfaces beside the sink such as marble: yes, no.

g. Spills on the kitchen floor: yes, no

h: interior and exterior surfaces of the refrigerator: yes, no
